# Identification of differentially expressed circRNAs and a novel hsa_circ_0000144 that promote tumor growth in gastric cancer

**DOI:** 10.1186/s12935-019-0975-y

**Published:** 2019-10-15

**Authors:** Jianming Wei, Jinmiao Wang, Xibo Gao, Feng Qi

**Affiliations:** 1 Department of General Surgery, Tianjin Medical University General Hospital, Tianjin, China; 2 Department of Pediatric Surgery, Tianjin Medical University General Hospital, Tianjin, China; 3 Department of Dermatology, Tianjin Children’s Hospital, Tianjin, China

**Keywords:** hsa_circ_0000144, circRNA–miRNA–mRNA regulatory network, Gastric cancer, Proliferation, Migration, Invasion

## Abstract

**Background:**

Circular RNAs (circRNAs) are involved in regulating tumor pathogenesis. The mechanism of circRNAs in gastric cancer (GC) is still unknown. Our study aimed to identify differentially expressed circRNAs and assess a novel circRNA (hsa_circ_0000144) in the proliferation, migration, and invasion in GC.

**Methods:**

Gene ontology (GO) enrichment and analyses of Kyoto Encyclopedia of Genes and Genomes (KEGG) pathways, pathway network, and the ceRNA regulatory network of hsa_circ_0000144 targeting miRNAs and mRNAs were performed with the help of bioinformatics using R language and Perl software. hsa_circ_0000144 expression and circRNA knockdown in GC cell lines were detected using quantitative PCR (qPCR) in vitro. Cell proliferation, migration, and invasion after circRNA knockdown were measured using the cell counting kit-8 assay and Transwell assay.

**Results:**

The circRNA expression profile GSE78092 downloaded from the Gene Expression Omnibus database included three GC patients and three normal tissues. Thirty-two differentially expressed circRNAs comprised six upregulated circRNAs and 26 downregulated circRNAs. In particular, the ErbB signaling pathway, neurotrophin signaling pathway, cellular senescence, and pathways in bladder cancer and GC played the most important roles in the pathway network. The expression of hsa_circ_0000144 was upregulated in GC cell lines. Hsa_circ_0000144 knockdown suppressed tumor growth in vitro.

**Conclusions:**

Hsa_circ_0000144 promotes GC cell proliferation, migration, and invasion, and the ceRNA regulatory network of hsa_circ_0000144 targeting miRNAs and mRNAs might be biomarkers for GC diagnosis and targeted therapy.

## Background

Gastric cancer (GC) is the fifth most common cancer and the third leading cause of cancer-related deaths [1]. In 2018, there were over 1,000,000 new cases and 783,000 deaths. Although many novel therapeutic pathways and diagnosis technologies have been used in GC, the clinical outcome of patients with GC remains very poor, with a 5-year overall survival of GC < 30% [2]. The mechanisms underlying GC pathogenesis need to be clarified to improve the clinical outcome.

Non-coding RNAs (ncRNAs) that regulate proliferation and invasion of GC cells have been evaluated in previous studies. MicroRNAs (miRNAs) and long ncRNAs (lncRNAs) play important roles in tumor biology [3–5]. Moreover, circular RNAs (circRNAs) are involved in various human diseases, particularly cancers [6–10].

Hsa_circ_0000144, which was generated from the back splicing of SLAMF6 first intron, is also known as circSLAMF6. It has been previously detected in bladder cancer. However, the underlying mechanism of hsa_circ_0000144 in GC is still unknown. In this study, we demonstrate that circSLAMF6 promotes tumor proliferation and invasion in GC and explore the regulatory network of circRNA targeting miRNA–mRNA. These data provide evidence for targeting circSLAMF6 to explore therapeutic measures and mechanisms underlying the pathogenesis of GC.

## Materials and methods

### ArrayStar microarray data

We used the keywords “gastric cancer circRNA” to search the National Centre of Biotechnology Information (NCBI) Gene Expression Omnibus database (GEO, https://www.ncbi.nlm.nih.gov/geo/), and the gene chip data sets GSE78092 (https://www.ncbi.nlm.nih.gov/gds/?term=GSE78092), submitted by Huang et al., were downloaded. GSE78092 contained six samples including three GC tissues and three normal tissues based on the GPL21485 platform of the ArrayStar human circular RNA microarray V2.0. The miRNA expression, clinical, and meta- and manifest data on GC were downloaded from The Cancer Genome Atlas (TCGA).

### Identification of differential circRNA expression

The circRNA expression data were converted using R language and Perl software. The circRNAs in these platforms were named according to international standard names. Differentially expressed circRNAs were identified by the limma package in the Bioconductor package (source (https://bioconductor.org/biocLite.R, biocLite ("limma")). P-values of 0.05 and log-fold change of 2 were considered evidence of significant difference. Volcano plots and heat maps were constructed using limma and pheatmap packages, respectively.

### miRNA target gene prediction and prognosis of miRNAs

We obtained the miRNAs targeting hsa_circ_0000144 in the circinteractome database (https://circinteractome.nia.nih.gov/bin/mirnasearch) and built an miRNA txt file. We then, downloaded four files, including miRDB.tsv, miRTarBase.tsv, and TargetScan.tsv, and identified miRNA targeted genes. Next, we extracted the expression and survival time of these miRNAs. The overall survival curves were analyzed using the R language survival package.

### Interaction competitive endogenous RNA (ceRNA) network of circRNA–miRNA–mRNA

Type and network txt files were designed. The interaction ceRNA network of circRNA–miRNA–mRNA was constructed using Cytoscape software 3.6.0 [11].

### GO enrichment and KEGG pathway analyses

GO enrichment and KEGG pathway analyses were performed using R language and Perl software. We installed the packages ("colorspace") ("dose"), biocLite ("DOSE") ("clusterProfiler"), and ("pathview").

### Cell culture and transfection

The GC cell line MGC-803 and normal cell line GES-1 were purchased from the Chinese Academy of Sciences (Shanghai, China). MGC-803 cells were cultured in F-12K and DMEM-H medium (Gibco, USA), respectively. All cells were cultured at 37 °C for 18 to 24 h in a humidified incubator containing 5% CO_2_. MGC-803 cells were transfected with plasmids using Lipofectamine 2000 reagent (Invitrogen, USA) based on the manufacturer's instructions. The expression of hsa_circ_0000144 in the transfected cells was detected by quantitative PCR (qPCR).

### CCK-8 cell proliferation assay

After 24 h of incubation, transfected cells were plated onto 96-well plates and cultured for 48 h. Every well contained 3000 cells. 3-(4,5-Dimethylthiazol-2-yl)-2,5-diphenyltetrazolium bromide (MTT) solution was added to each well, and cell viability was assessed by measuring the absorbance at 450 nm.

### Transwell migration and invasion assay

To examine cell migration and invasion ability, we conducted the Transwell assay according to the manufacturer’s instructions. Cells were incubated for 24 h. Three microscopy fields were randomly selected to acquire images.

### Real-time qRT-PCR analysis

Total RNA was extracted using TRIzol reagent (Invitrogen, USA) and synthesized into cDNA using M-MLV reverse transcriptase (TaKaRa Bio, Japan) following the manufacturer’s instructions. qRT-PCR was performed using SYBR Green assay (Roche, Switzerland). Glyceraldehyde 3-phosphate dehydrogenase (GAPDH) and U6 were used as controls. The primer sequences were:

GAPDH-S: 5′-ACACCCACTCCTCCACCTTT-3′

GAPDH-AS: 5′-TTACTCCTTGGAGGCCATGT-3′

Hsa_circ_0000144-S: 5′-GAGTGTTGGCCTGTCCTCAA-3′

Hsa_circ_0000144-AS: 5′-TTGTGCCCAGTTGCCTGTAT-3′

### Statistical analyses

circRNA expression data analysis was performed using GraphPad Prism 6.0 software, and survival analysis was performed using the R language survival package. P < 0.05 was considered significant.

## Results

### Identification of differential expression of circRNAs

**Fig. 1:**
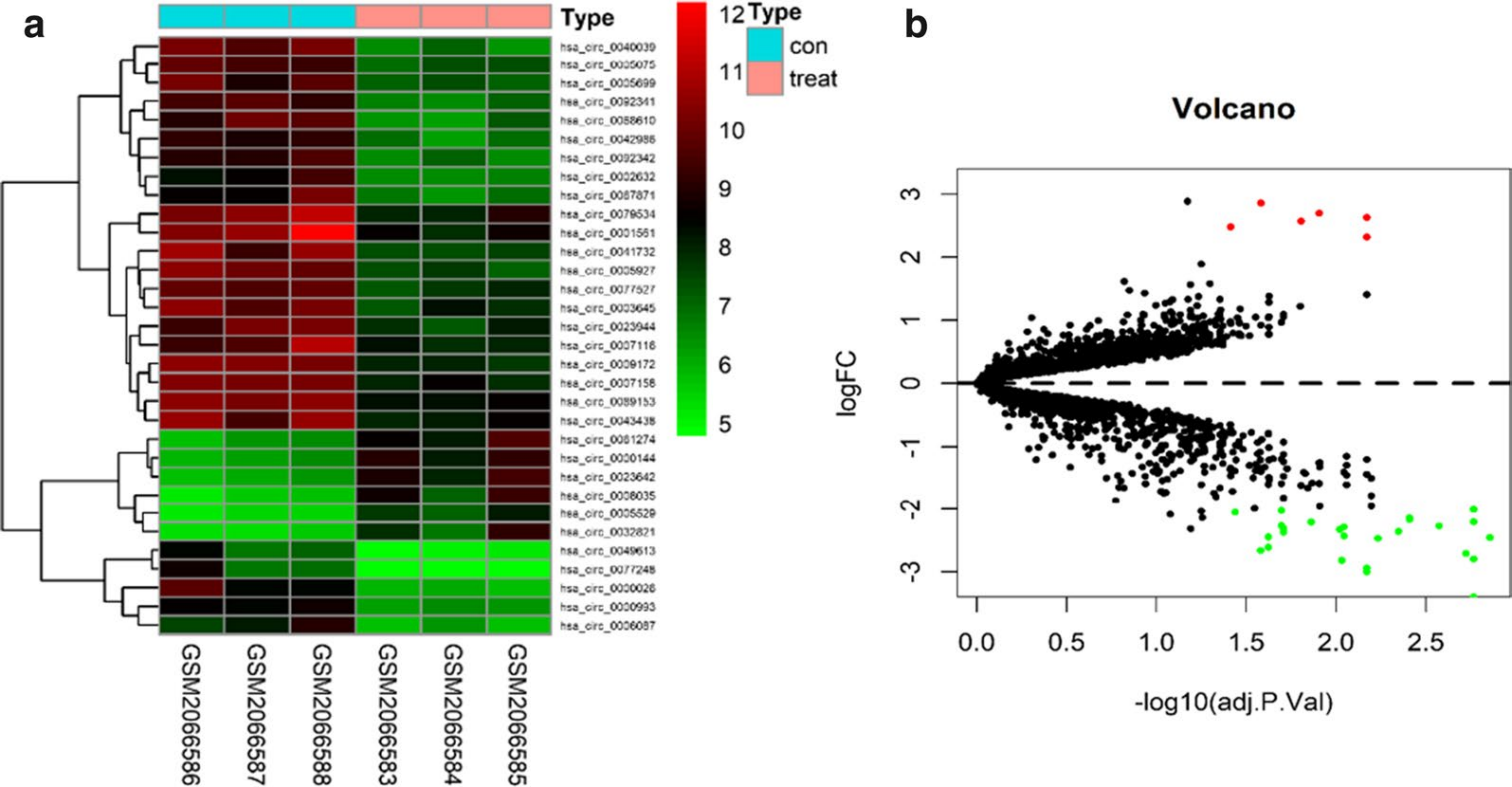
Differential expression of circRNAs from GEO database GSE78092 samples. **a** Hierarchical clustering heat map of differential expression of circRNAs. **b** Volcano plot. Red represents upregulated circRNAs and green represents downregulated circRNAs

We analyzed the data GSE78092 from the GEO database containing three GC tissues and three adjacent normal tissues. A total of 32 circRNAs were differentially expressed in the GC samples compared to the normal samples. Among the differentially expressed circRNAs, 26 were downregulated and six were upregulated. These results are presented in the volcano plot and heat map (Fig. 1). The top 12 circRNAs with the most significant expression were hsa_circ_0009172, hsa_circ_0089153, hsa_circ_0005927, hsa_circ_0040039, hsa_circ_0000993, hsa_circ_0092341, hsa_circ_0005075, hsa_circ_0077527, hsa_circ_0007158, hsa_circ_0042986, hsa_circ_0092342, and hsa_circ_0000144.

### miRNAs and target gene prediction

**Fig. 2:**
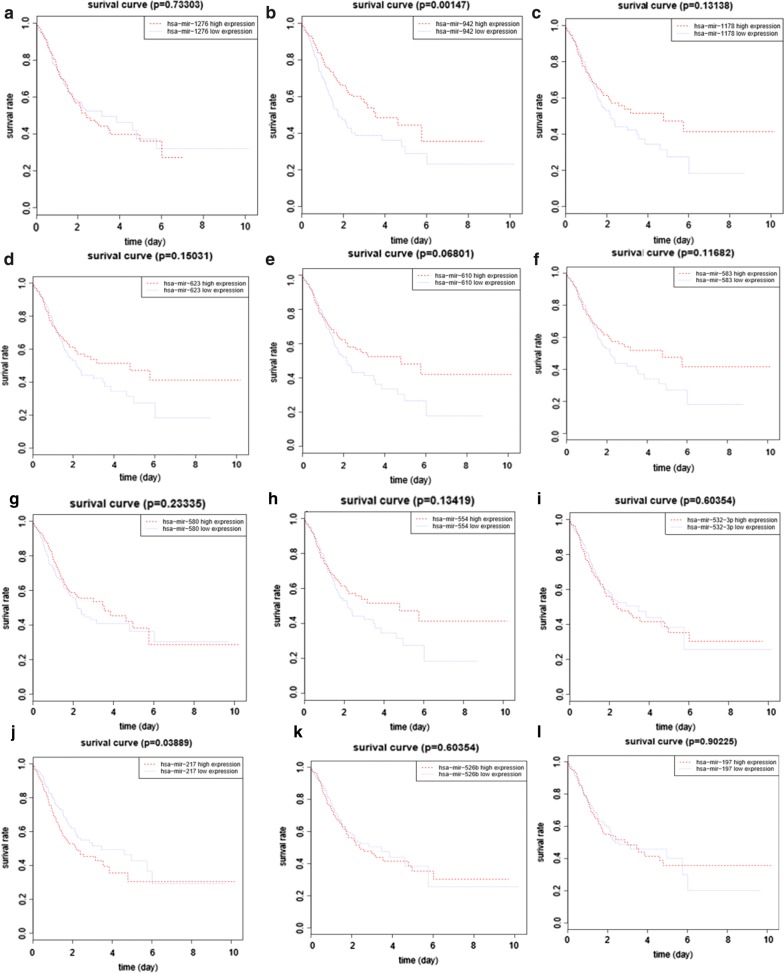
Relationship between expression of miRNAs targeting hsa_circ_0000144 and survival. **a** hsa-mir-1276, **b** hsa-mir-942, **c** hsa-mir-1178, **d** hsa-mir-623, **e** hsa-mir-610, **f** hsa-mir-583, **g** hsa-mir-580, **h** hsa-mir-554, **i** hsa-mir-532-3p, **j** hsa-mir-217, **k** hsa-mir-526b, and **l** hsa-mir-197. Red line represents high expression, and blue line represents low expression

We used the keyword “hsa_circ_0000144” in the circinteractome database (https://circinteractome.nia.nih.gov/bin/mirnasearch) and obtained the miRNAs that could bind to the circRNA hsa_circ_0000144. These included hsa-mir-1178, hsa-mir-1276, hsa-mir-197, hsa-mir-217, hsa-mir-485-3p, hsa-mir-502-5p, hsa-mir-526b, hsa-mir-532-3p, hsa-mir-554, hsa-mir-580, hsa-mir-583, hsa-mir-610, hsa-mir-623, and hsa-mir-942. The relationship of miRNAs targeting genes is listed in Table 1. The relationship of miRNA expression with survival is depicted in Fig. 2; hsa-mir-217 and hsa-mir-942 were significantly associated with survival (P < 0.05).

**Table 1:** miRNAs targeted genes prediction using perl language program

hsa-miR-1276	DPP6 TPM3 LAMP2 DDX5 NOL11 INTS2 STARD3NL USP44 GPBP1 VANGL2
hsa-miR-217	TNFRSF21 GPC5 TCF7L2 SIRT1 PPM1D LMLN DACH1 MAP1B SNRNP27 KRAS EZH2 FOXO3 NR4A2 ADSS
hsa-miR-623	PPP6R3 RANBP1 SRPX2 ATF6B NUPL2 CACNA1C IRF2BP2 NUDT7 PGRMC2 TMEM156 SOD2
hsa-miR-610	CYBRD1 DNAJC10 FEN1 CENPA ELK3
hsa-miR-583	PSME3 PNN HNRNPC YWHAH PRRG4 PIM1 TFAP4 YOD1
	IGF1R GNAI2 ENTPD4 B4GALT1 ARF6 CBX5 SON
	SIVA1 FAXC IPMK FAM53B ARID1A PLAGL2 GCC1
hsa-miR-532-3p	SVOP RAPGEF6 ATP2B1 SREBF1 KPNA6 AP2B1 MAFK
hsa-miR-554	NONO BTRC EIF1
hsa-miR-502-5p	MACROD2 B3GALT5 RAB1B DAZAP2 ODF4 H1F0nn
hsa-miR-485-3p	SRSF2 IDS KPNA2 NFYB ARID1A SLC40A1 NRF1 PCGF3

### Constructed interaction of the circRNA–miRNA–mRNA ceRNA network

**Fig. 3:**
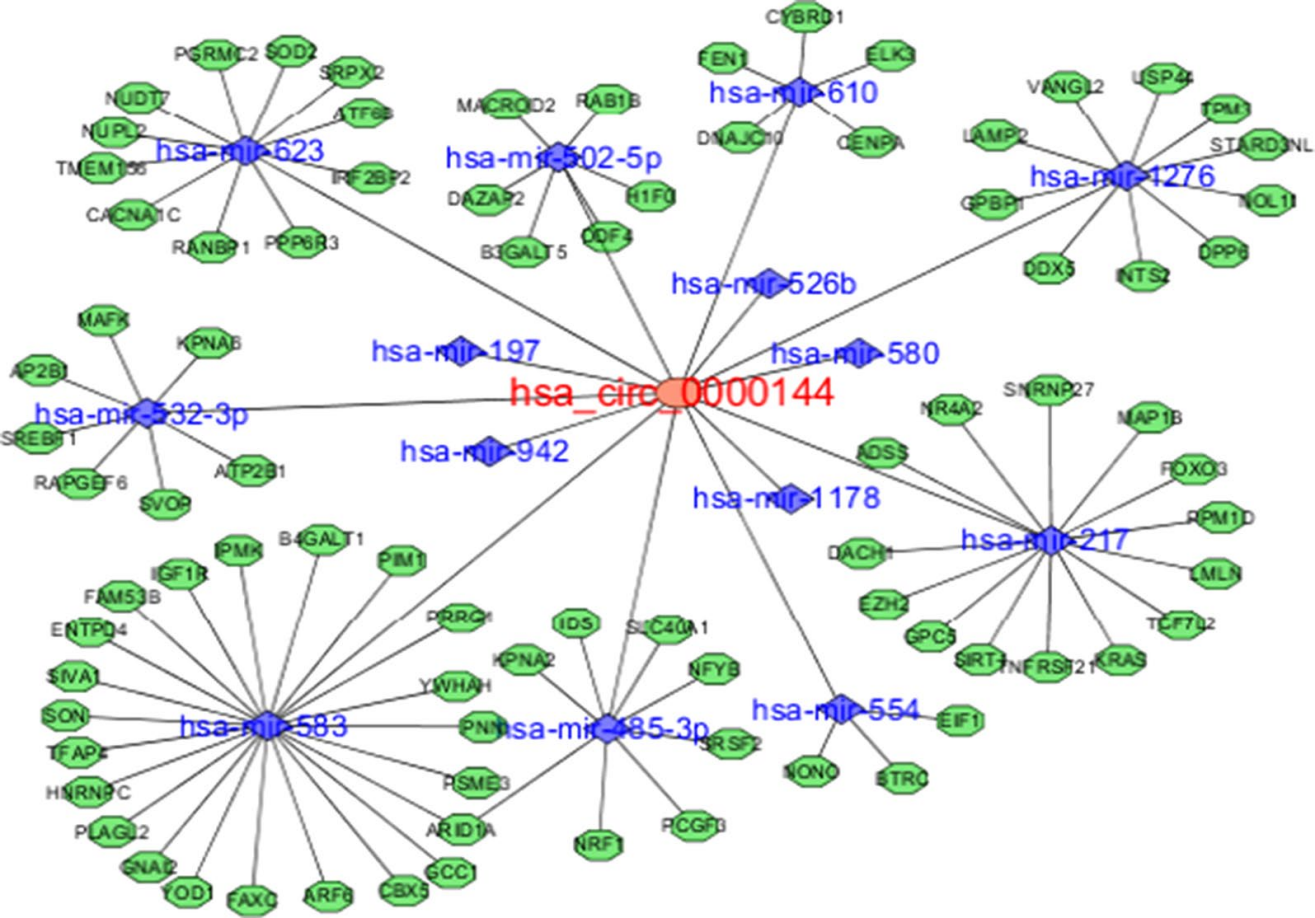
ceRNA network of circRNA hsa_circ_0000144 targeting miRNAs and mRNAs. Red represents circRNA hsa_circ_0000144, blue represents miRNAs, and green represents genes

We designed the type and network txt files. The circRNA–miRNA–mRNA regulatory network was constructed using Cytoscape software 3.6.0. Hsa_circ_0000144 interacted with miRNAs and genes (Fig. 3).

### GO enrichment and KEGG pathway analyses

**Fig. 4:**
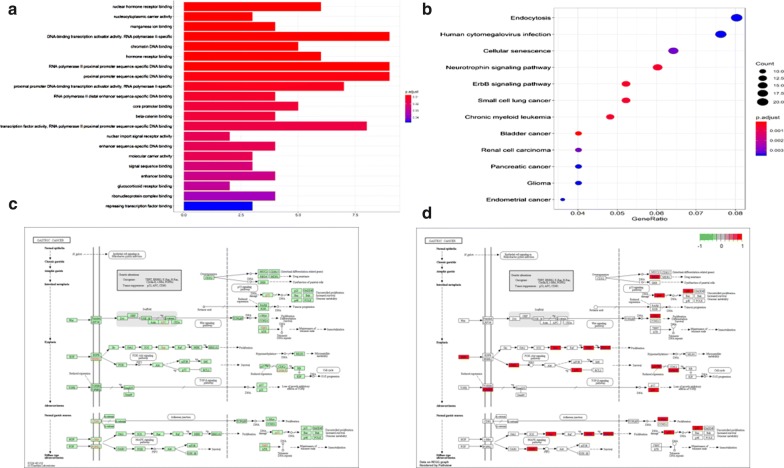
GO enrichment and KEGG pathway analyses. **a** Significant GO enrichment items. **b** Gene ratio and KEGG pathway items. **c** KEGG pathway in GC (green represents downregulated genes). **d** KEGG pathway in GC (red represents upregulated genes)

GO enrichment and KEGG pathway analyses were performed using R language and Perl software. GO functional enrichment analysis of genes with P < 0.05 was obtained. The KEGG result was shown in Additional file 1. Nuclear hormone receptor binding was the most significant enrichment (Fig. 4a). The signaling pathways of differentially expressed circRNAs were mainly enriched in the ErbB signaling pathway, neurotrophin signaling pathway, cellular senescence, and pathways involved in bladder cancer and GC (Fig. 4b).

### Expression of hsa_circ_0000144 in GC tumor cell lines and normal cell lines

**Fig. 5:**
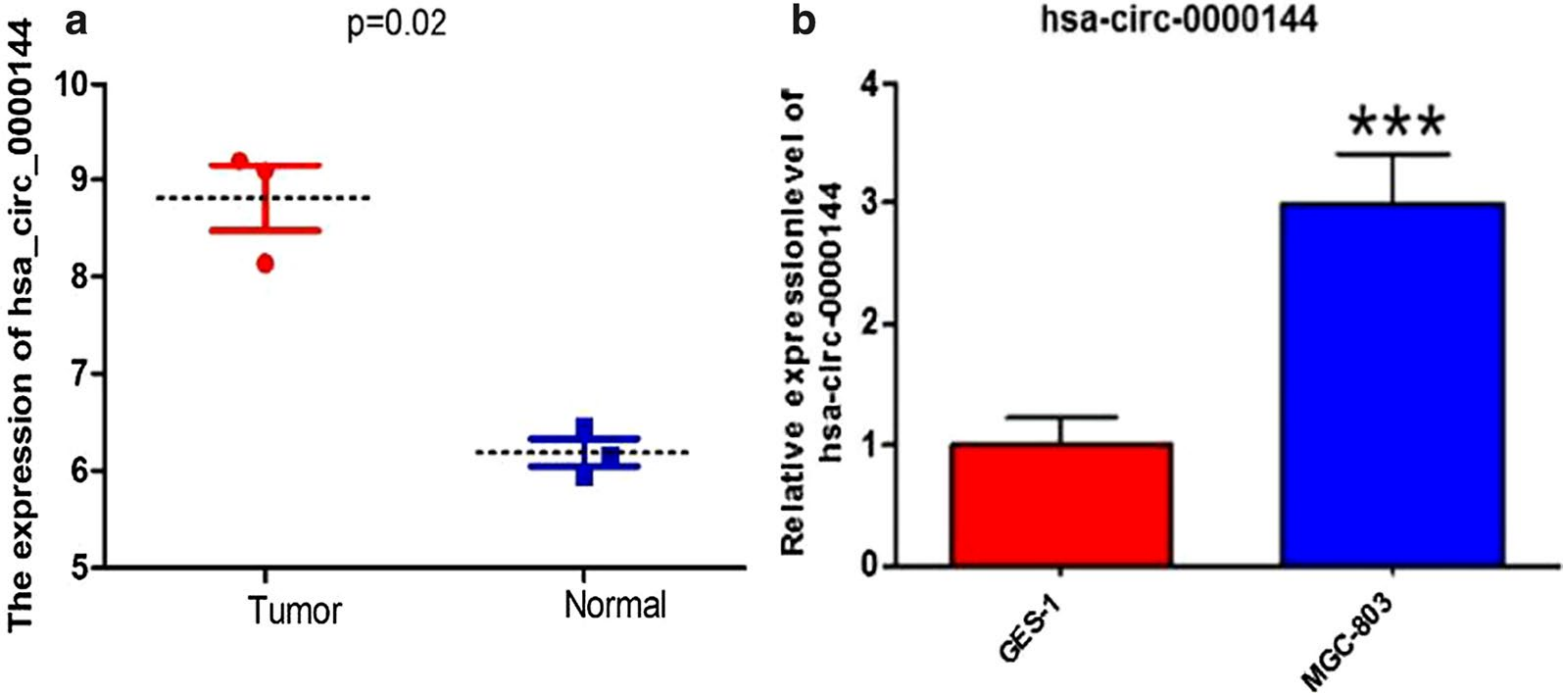
Expression of hsa_circ_0000144 in GC. **a** CircRNA hsa_circ_0000144 was upregulated in GSE78902 samples from the GEO database. **b** CircRNA hsa_circ_0000144 showed higher expression in MGC-803 GC cells compared to normal GES-1 cells

Hsa_circ_0000144 was highly expressed in GSE78902 samples from the GEO database (Fig. 5a). To further verify the results, we chose two cell lines and found that the expression of hsa_circ_0000144 was upregulated in MGC-803 GC cancer cells lines compared to GES-1 normal cells (Fig. 5b).

### Expression of hsa_circ_0000144 in different groups

**Fig. 6:**
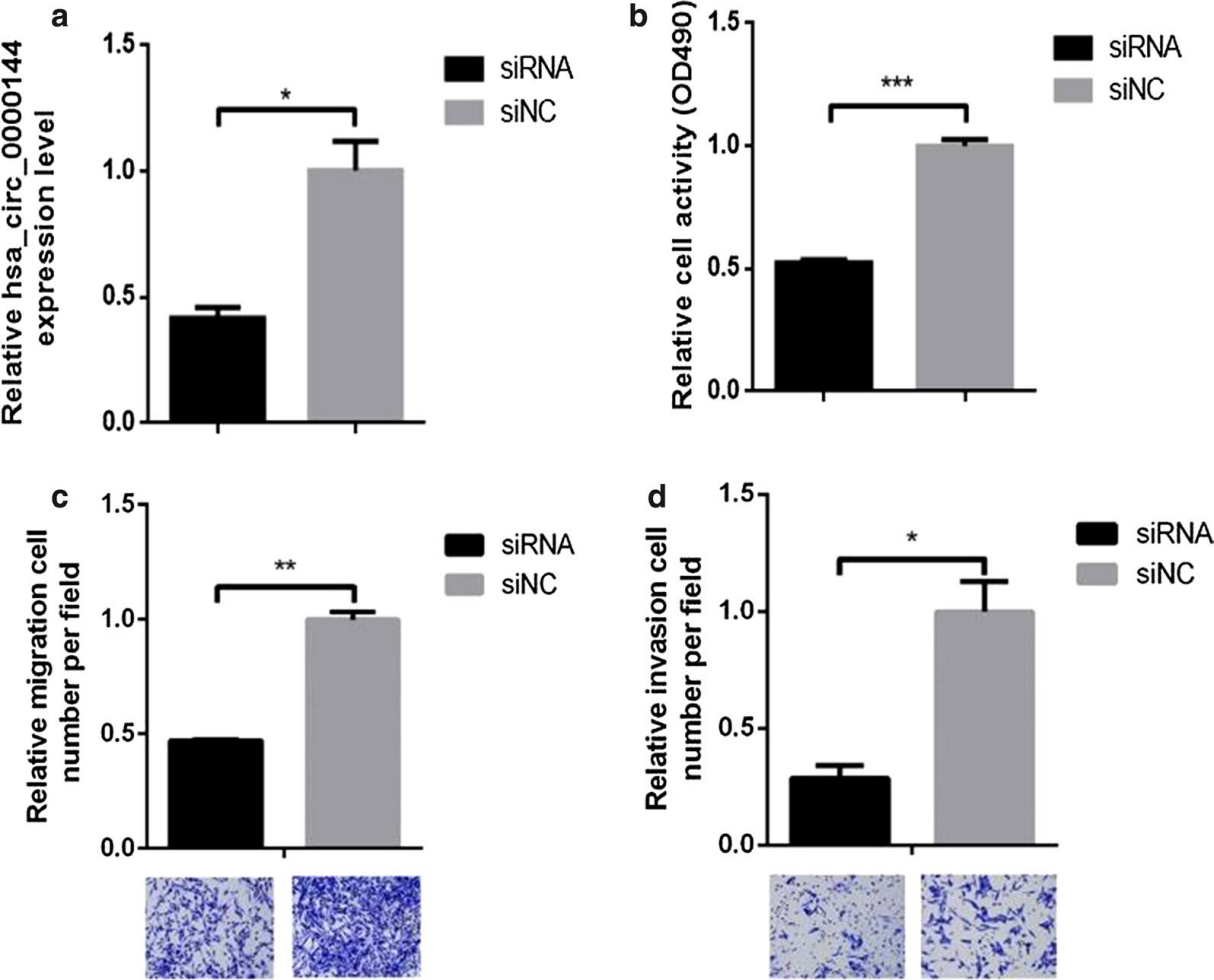
CircRNA hsa_circ_0000144 promotes proliferation, migration, and invasion. **a**, **b** Hsa_circ_0000144 knockdown inhibits the proliferation of MGC-803 cells. **c**, **d** Hsa_circ_0000144 knockdown inhibits the migration and invasion of MGC-803 cells

To assess the interference efficiency of different siRNA sequences on hsa_circ_0000144 expression, the expression of hsa_circ_0000144 mRNA was detected by qPCR after transfection of MGC-803 cells with different siRNA sequences. Hsa_circ_0000144 expression with small interfering (si)RNA was lower than with siNC (Fig. 6a).

### SiRNA-hsa_circ_0000144 inhibits proliferation in AGS cell lines

The CCK8 assay was used to evaluate the effect of hsa_circ_0000144 on proliferation. SiRNA-hsa_circ_0000144 markedly inhibited cell proliferation (Fig. 6b).

### Hsa_circ_0000144 knockdown inhibits migration and invasion of MGC-803 cells

Transwell migration and invasion assays were performed to further examine the effect of hsa_circ_0000144 on GC metastasis. Hsa_circ_0000144 significantly affected cell invasion and migration (Fig. 6c, d).

## Discussion

Globally, GC is a common disease and is the second leading cause of cancer-related deaths [12, 13]. The mechanism underlying the pathogenesis of GC is currently unknown. The regulatory mechanism of GC should be clarified. CircRNAs play different roles in different tissues and cell types [14]. The emerging function of circRNAs in tumorigenesis has garnered recent interest. Herein, we explored the role of hsa_circ_0000144, derived from the *SLAF6* gene, in the pathogenesis of GC.

We analyzed data on GC circRNAs from the GEO database using R software and bioinformatics. Thirty-two differentially expressed circRNAs were identified, including six upregulated circRNAs and 26 downregulated circRNAs. Hsa_circ_0000144, hsa_circ_0005529, hsa_circ_0023642, hsa_circ_0061274, hsa_circ_0008035, and hsa_circ_0032821 were upregulated. Hsa_circ_0000144 was the most significantly different circRNA expressed.

From the circinteractome database, we obtained 14 miRNAs targeting hsa_circ_0000144 and tested them using the miRNA expression data of the TCGA database. The expression of hsa-mir-217 and hsa-mir-942 was associated with increased survival in GC (Fig. 2b, j). Previous studies reported that miR-217 has different roles in tumor proliferation, migration, and invasion [15–18]. Presently, the high expression of miR-942 was associated with better prognosis in GC (Fig. 2b). Conversely, another study showed that miR-942 promoted tumor pathogenesis [19]. This dichotomy underscores the need to further assess the mechanism underlying the role of miR-942 in GC.

The ceRNA of the lncRNA–miRNA–mRNA network affects tumor regulation. CircRNAs can function as ceRNAs in tumor biology [20, 21]. Recent studies have indicated that circRNAs may act as ceRNAs to sequester miRNAs of a particular family, thereby serving as competitive inhibitors that suppress the ability of a miRNA to bind its mRNA targets [22, 23]. A previous study demonstrated that hsa_circ_0000144 serves as an endogenous sponge for miR-217 and validated that hsa_circ_0000144 directly binds to miR-217 using a luciferase reporter assay in bladder cancer cells [18].

Presently, we analyzed the ceRNA network of circRNA-hsa_circ_0000144 targeting miRNA–mRNA. We found that hsa_circ_0000144 barely interacted with miRNAs and mRNAs (Fig. 3). To detect the gene signal and pathway networks, GO enrichment and KEGG pathway analyses were performed. GO enrichment analysis revealed that genes that interacted with circRNAs by miRNAs were mainly involved in pathways related to nuclear hormone receptor binding, which had the most significant enrichment (Fig. 4a). The signaling pathways of differentially expressed circRNAs were mainly enriched in the ErbB signaling, neurotrophin signaling, and cellular senescence pathways, and pathways in bladder cancer and GC (Fig. 4b). Recent observations indicate that the ErbB signaling pathway contributes to pancreatic tumorigenesis [24, 25]. ErbB signaling is a prerequisite for maintenance of the intestinal epithelium following injury and tumor formation [26, 27]. Neurotrophins are a family of closely structurally related growth factors [28], which include nerve growth factor, brain-derived neurotrophic factor, neurotrophin 3 (NT-3), and NT-4/5. Neurotrophins exert a range of effects on cell proliferation and migration in non-neuronal cells as well as in cancer cells [29]. Recent findings have revealed that neurotrophin signaling pathways are also a driver of tumor neurogenesis, via the stimulation of NGF receptors on nerve endings.

Recent evidence has also demonstrated that circRNAs affect functions in cancer [30, 31]. Although the mechanism of circRNAs has been reported in many cancers, its mechanism underlying carcinogenesis and cancer progression in GC has not been fully elucidated. In the present study, we identified a novel circRNA, hsa_circ_0000144, from the GEO database, which was increased in GSE78092 (Fig. 1). We detected the expression of circRNA hsa_circ_0000144 in MGC-803 GC cells and GES-1 normal cells and found that hsa_circ_0000144 was significantly upregulated in MGC-803 cells. Further analysis of the GC phenotype in vivo showed that hsa_circ_0000144 knockdown suppressed bladder cancer cell proliferation, invasion, and migration in vitro (Fig. 6b–d).

## Conclusion

Hsa_circ_0000144 promotes GC cell proliferation and invasion. The ceRNA regulatory network of hsa_circ_0000144 targeted miRNAs and mRNAs, indicating the potential value as biomarkers for GC diagnosis and targeted therapy.

## Supplementary information

**Additional file 1.** KEGG pathway analysis of genes differentially expressed circRNAs targeting.

## Data Availability

Not applicable.

## References

[1] Bray F, Ferlay J, Soerjomataram I, Siegel RL, Torre LA, Jemal A. Global Cancer Statistics 2018: GLOBOCAN estimates of incidence and mortality worldwide for 36 cancers in 185 countries. CA Cancer J Clin. 2018;0:23–31. 10.3322/caac.21492 30207593

[2] Allemani C, Weir HK, Carreira H, Harewood R, Spika D, Wang XS (2015). Global surveillance of cancer survival 1995–2009: analysis of individual data for 25,676,887 patients from 279 population-based registries in 67 countries (CONCORD-2). Lancet.

[3] Wang QX (2015). Altered MiRNA expression in gastric cancer: a systematic review and meta-analysis. Cell Physiol Biochem.

[4] Huang YK, Yu JC (2015). Circulating microRNAs and long non-coding RNAs in gastric cancer diagnosis: an update and review. World J Gastroenterol.

[5] Virgilio E (2018). Long non-coding RNAs in the gastric juice of gastric cancer patients. Pathol Res Pract.

[6] Rong D, Tang W, Li Z, Zhou J, Shi J, Wang H, Cao H (2017). Novel insights into circular RNAs in clinical application of carcinomas. Onco Targets Ther.

[7] Chen Y (2019). Circular RNA circAGO2 drives cancer progression through facilitating HuR-repressed functions of AGO2–miRNA complexes. Cell Death Differ.

[8] Li Y (2017). CircHIPK3 sponges miR-558 to suppress heparanase expression in bladder cancer cells. EMBO Rep.

[9] Han D, Li J, Wang H, Su X, Hou J, Gu Y (2017). Circular RNA circMTO1 acts as the sponge of microRNA-9 to suppress hepatocellular carcinoma progression. Hepatology.

[10] Funaki NO, Tanaka J, Kasamatsu T, Ohshio G, Hosotani R, Okino T, Imamura M (1996). Identification of carcinoembryonic antigen mRNA in circulating peripheral blood of pancreatic carcinoma and gastric carcinoma patients. Life Sci.

[11] Franz M, Lopes CT, Huck G, Dong Y, Sumer O, Bader GD (2016). Cytoscape.js: a graph theory library for visualisation and analysis. Bioinformatics.

[12] Guo J, Miao Y, Xiao B, Huan R, Jiang Z, Meng D, Wang Y (2006). Differential expression of microRNA species in human gastric cancer versus non-tumorous tissues. J Gastroenterol Hepatol..

[13] Li T, Mo X, Fu L, Xiao B, Guo J (2016). Molecular mechanisms of long noncoding RNAs on gastric cancer. Oncotarget..

[14] Starke S, Jost I, Rossbach O (2015). Exon circularization requires canonical splice signals. Cell Rep.

[15] Lin Y, Cheng K, Wang T, Xie Q, Chen M, Chen Q, Wen Q (2017). miR-217 inhibits proliferation, migration, and invasion via targeting AKT3 in thyroid cancer. Biomed Pharmacother.

[16] Yu B, Ye X, Du Q, Zhu B, Zhai Q, Li XX (2017). The Long Non-coding RNA CRNDE promotes colorectal carcinoma progression by competitively binding miR-217 with TCF7L2 and enhancing the Wnt/β-catenin signaling pathway. Cell Physiol Biochem.

[17] Zhao WG, Yu SN, Lu ZH, Ma YH, Gu YM, Chen J (2010). The miR-217 microRNA functions as a potential tumor suppressor in pancreatic ductal adenocarcinoma by targeting KRAS. Carcinogenesis.

[18] Huang W, Lu Y, Wang F, Huang X, Yu Z (2018). Downregulation of circular RNA hsa_circ_0000144 inhibits bladder cancer progression via stimulating miR-217 and suppressing RUNX2 expression. Gene.

[19] Shan Z, An N, Qin J, Yang J, Sun H, Yang W (2018). Long non-coding RNA Linc00675 suppresses cell proliferation and metastasis in colorectal cancer via acting on miR-942 and Wnt/β-catenin signaling. Biomed Pharmacother.

[20] Qi X (2015). ceRNA in cancer: possible functions and clinical implications. J Med Genet.

[21] Ergun S, Oncocers OS (2015). ceRNA-mediated cross-talk by sponging miRNAs in oncogenic pathways. Tumour Biol.

[22] Chen Y, Li C, Tan C, Liu X (2016). Circular RNAs: a new frontier in the study of human diseases. J Med Genet.

[23] Zhao ZJ, Shen J (2017). Circular RNA participates in the carcinogenesis and the malignant behavior of cancer. RNA Biol.

[24] Ardito CM, Gruner BM, Takeuchi KK (2012). EGF receptor is required for KRAS-induced pancreatic tumorigenesis. Cancer Cell.

[25] Navas C, Hernandez-Porras I, Schuhmacher AJ (2012). EGF receptor signaling is essential for k-ras oncogene-driven pancreatic ductal adenocarcinoma. Cancer Cell.

[26] Lee D (2009). Tumor-specific apoptosis caused by deletion of the ERBB3 pseudo-kinase in mouse intestinal epithelium. J Clin Invest.

[27] Roberts RB (2002). Importance of epidermal growth factor receptor signaling in establishment of adenomas and maintenance of carcinomas during intestinal tumorigenesis. Proc Natl Acad Sci USA.

[28] Skaper SD (2012). The neurotrophin family of neurotrophic factors: an overview. Methods Mol Biol.

[29] Bradshaw RA, Pundavela J, Biarc J, Chalkley RJ, Burlingame AL, Hondermarck H (2015). NGF and ProNGF: regulation of neuronal and neoplastic responses through receptor signaling. Adv Biol Regul.

[30] Li F, Zhang L, Li W (2015). Circular RNA ITCH has inhibitory effect on ESCC by suppressing the Wnt/β-catenin pathway. Oncotarget.

[31] Chen X, Fan S, Song E (2016). Noncoding RNAs: new players in cancers. Adv Exp Med Biol.

